# Leukocyte telomere length is associated with elevated plasma glucose and HbA1c in young healthy men independent of birth weight

**DOI:** 10.1038/s41598-019-43387-0

**Published:** 2019-05-21

**Authors:** L. G. Grunnet, K. Pilgaard, A. Alibegovic, C. B. Jensen, L. Hjort, S. E. Ozanne, M. Bennett, A. Vaag, C. Brøns

**Affiliations:** 1 Department of Endocrinology, Diabetes & Metabolism, Rigshospitalet, Copenhagen, Denmark; 2 The Danish Diabetes Academy, Odense, Denmark; 3 Nordsjællands Hospital, Hillerød, Denmark; 4 Steno Diabetes Center Copenhagen, Gentofte, Denmark; 5 Novo Nordisk, Søborg, Denmark; 6 University of Cambridge Metabolic Research Laboratories and MRC metabolic Diseases Unit, Cambridge, UK; 7 Division of Cardiovascular Medicine, Addenbrooke’s Hospital, Cambridge, UK; 8 Cardiovascular and Metabolic Disease (CVMD) Translational Medicine Unit, Early Clinical Development, IMED Biotech Unit, AstraZeneca, Gothenburg Sweden

**Keywords:** Genetic markers, Endocrine system and metabolic diseases

## Abstract

Telomeres are protein-bound regions of repetitive nucleotide sequences (TTAGGG) at the end of human chromosomes, and their length is a marker of cellular aging. Intrauterine growth restriction is associated with shorter blood cell telomeres at birth and individuals with type 2 diabetes have shorter telomeres. Individuals with a low birth weight (LBW) have an increased risk of metabolic disease and type 2 diabetes. Therefore, we aimed to investigate the relationship between birth weight and telomere length and the association between birth weight, telomere length and cardiometabolic phenotype in adulthood. Young, healthy men with LBW (n = 55) and normal birth weight (NBW) (n = 65) were examined including blood pressure, blood samples and body composition. Leukocyte telomere length was determined using a high-throughput qPCR method. The LBW men were more insulin resistant as determined by the HOMA-IR index. There was no difference in telomere length between LBW and NBW subjects. When adjusting for birth weight and cohort effect, significant negative associations between telomere length and fasting glucose (P = 0.003) and HbA1c (P = 0.0008) were found. In conclusion, no significant difference in telomere length was found between LBW and NBW men. The telomere length was negatively associated with glucose concentrations and HbA1c levels within the normal non-diabetic range independent of birth weight.

## Introduction

Telomeres are protein-bound regions of repetitive nucleotide sequences (TTAGGG) at the end of human chromosomes. They maintain genomic stability by protecting the ends of the chromosome from deterioration^1^. DNA damage responses are activated when telomeres are shortened to a critical length, causing cellular senescence. Telomeres are longest at birth and shorten in most somatic cells postnatally due to both cell division and oxidative stress^2^. In telomerase negative cells telomere shortening is irreversible and thus telomere length can be used as a biomarker for biological cellular aging^3^. In support of this, a number of studies have demonstrated that telomere shortening is affected by cell age^4,5^. Shorter telomeres have also been associated with a number of factors including male gender^6^, Caucasian race^4^, inflammation^7^, body composition and markers of glucose metabolism^8^. Results from a meta-analysis indicate that shortened leucocyte relative telomere length is significantly associated with type 2 diabetes and leucocyte telomere length with stroke and myocardial infarction^9,10^.

Early-life environmental exposures impact on risk of developing cardiometabolic disease later in life as suggested by Hales and Barker^11^. It has been shown that fetal undernutrition resulting in a low birth weight (LBW) for gestational age, gives rise to a phenotype associated with increased risk of type 2 diabetes and cardiovascular disease, and we and others have demonstrated multiple metabolic derangements in healthy young men born with LBW^12–14^. Similarly, offspring of women with gestational diabetes mellitus (GDM) thus exposed to fetal overnutrition, exhibit increased risk of type 2 diabetes in adulthood^15^. Our understanding of mechanisms underlying the association between fetal exposures and later risk of disease is not comprehensive. Several potential molecular mechanisms have been suggested, including accelerated cellular ageing, as it appears to play a role in disease progression. An adverse intrauterine environment may thus contribute to the formation of short telomeres giving rise to a senescent phenotype in the adulthood. Studies have shown that maternal stress and smoking during pregnancy is positively associated with shortened telomeres in the offspring^16,17^ and poor fetal growth has been shown to be associated with shorter telomeres in newborns^18,19^ as well as in adolescents^20^. However, a recent study found no increased acceleration of telomere shortening in adult women born small for gestational age^21^, and another study found no evidence of telomere abnormalities and oxidative DNA damage in adult offspring of type 1 diabetes mothers as compared to control individual^22^.

The aim of the present study was to determine whether fetal malnutrition, as defined by a low birth weight, is associated with telomere shortening in a cohort of young healthy men, and to examine whether telomere length is associated with markers of cardiometabolic traits.

## Results

### Characteristics of the study participants

The participants born with a LBW had a median birth weight that was 1180 g lighter (P < 0.0001) than NBW individuals (2700 (2500–2800) vs. 3880 (3800–4000) g). As shown in Table 1, the LBW men had significantly shorter adult height (P < 0.0001), lower lean mass (P = 0.0008), elevated fasting plasma glucose levels (P = 0.01) and evidence of insulin resistance (P = 0.04) as measured by the homeostatic model assessment of insulin resistance (HOMA-IR), predominantly reflecting insulin action in the liver, compared with NBW participants. Furthermore, there was a tendency towards higher waist-hip ratio (P = 0.06) and increased fasting insulin levels (P = 0.09) in the LBW men (Table 1). There were no other differences between the two groups.

**Table 1:** Characteristics of the study participants.

	LBW (n = 55)	NBW (n = 65)	*P*-value
Age (years)	23.3 (2.3)	23.0 (2.5)	0.48
Weight (kg)	76.6 (11.4)	79.1 (10.9)	0.21
Height (cm)	179.1 (57.2)	184.4 (68.0)	<*0.0001*
BMI (kg/m^2^)	23.9 (3.5)	23.3 (3.1)	0.34
Waist-hip ratio	0.87 (0.05)	0.85 (0.07)	0.06
Lean mass (kg)^#^	56.5 (5.7)	60.7 (6.6)	*0.0008*
Fat mass trunk (kg)^*^	6.7 (4.8–11.3)	6.9 (5.3–9.6)	0.73
Fat mass total (kg)	16.3 (8.2)	15.6 (7.2)	0.60
Fat %^#^	18.7 (8.3)	16.8 (7.6)	0.25
SBP (mmHg)	128 (14)	127 (15)	0.89
DBP (mmHg)	69 (9)	70 (8)	0.52
F-p-glucose (mM)	5.5 (0.5)	5.3 (0.5)	*0.01*
F-p-insulin (pmol/l)^*^	33.7 (22.8–50.3)	27.1 (21.2–37.9)	0.09
HbA1c (%)^*^	5.2 (5.0–5.4)	5.1 (5.0–5.3)	0.30
HOMA-IR^*^	1.22 (0.85–1.78)	0.92 (0.72–1.33)	*0.04*
HOMA-B%^*^	49.7 (26.1–64.2)	47.2 (35.4–62.3)	0.72
FFA basal (µmol/l)^*^	363 (295–521)	340 (241–475)	0.18
Total cholesterol (mmol/l)	4.17 (1.03)	4.0 (0.82)	0.34
HDL (mmol/l)	1.22 (0.34)	1.28 (0.27)	0.31
LDL (mmol/l)	2.36 (0.73)	2.30 (0.74)	0.69
VLDL (mmol/l)^*^	0.40 (0.30–0.60)	0.40 (0.30–0.50)	0.63
Triglyceride (mmol/l)^*^	0.90 (0.70–1.26)	0.89 (0.69–1.10)	0.38
Leukocytes (10^−9^)^*^	5.5 (5.0–6.5)	5.4 (4.8–6.4)	0.60

Data are presented as mean (SD) and median (interquartile range) for normally and non-normally distributed variables, respectively, *Wilcoxon test, ^#^n = 46 LBW and 52 NBW.

### Telomere length and its association to phenotypic characteristics

**Figure 1:**
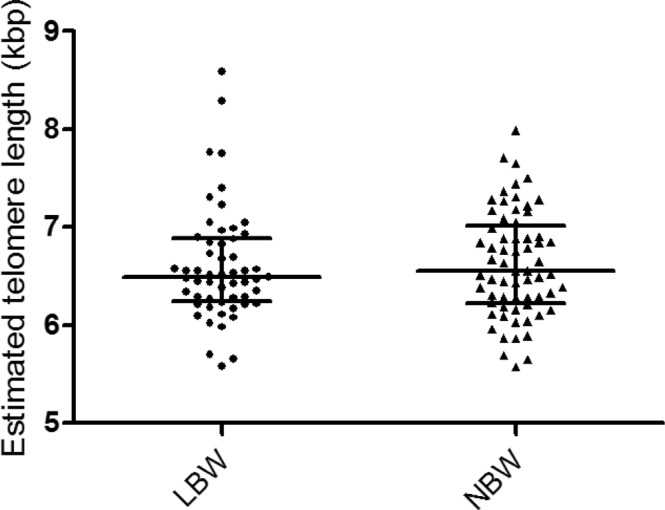
Estimated telomere length in LBW and NBW participants.

There was no difference in leukocyte telomere length between LBW and NBW participants (6.49 (6.24–6.88) vs 6.55 (6.23–6.99) kbp, P = 0.66) (Fig. 1).

In participants born with LBW, telomere length showed a positive association with systolic blood pressure (P = 0.05) and borderline significant positive association with diastolic blood pressure (P = 0.06) (Table 2). In addition, telomere length was negatively associated with fat% (P = 0.02), fasting plasma glucose (P = 0.002) and a tendency towards a negative association with HbA1c (P = 0.09) was observed. Among NBW participants, there was a negative association between telomere length and HbA1c (P = 0.002) only. No associations to the blood lipid profile or obesity measurements were found in any of the birth weight groups.

**Table 2:** Associations between telomere length and phenotypic characteristics.

	LBW (n = 55)		NBW (n = 65)	
	β (95% CI)	*p*-value	β (95% CI)	*p*-value
BMI (kg/m^2^)	−0.03 (−0.07; 0.02)	0.20	−0.02 (−0.06; 0.03)	0.51
Waist-hip ratio	1.21 (−1,88; 4.30)	0.44	1.12 (−0.94; 3.18)	0.28
Lean mass (g)*	−0.001 (−0.004; 0.02)	0.38	−0.001 (−0.004; 0.001)	0.26
Fat mass trunk (g)*	−0.002 (−0.005; 0.002)	0.38	−0.002 (−0.005; 0.002)	0.35
Fat mass total (g)*	−0.001 (−0.003; 0.001)	0.36	−0.009 (0.003; 0.001)	0.36
Fat %	−0.02 (−0.04; −0.004)	*0.02*	−0.001 (−0.02; 0.02)	0.90
SBP (mmHg)	0.01 (0.00003; 0.02)	*0.05*	0.003 (−0.01; 0.01)	0.52
DBP (mmHg)	0.02 (−0.00007; 0.04)	0.06	0.002 (−0.02; 0.02)	0.78
F-p-glucose (mM)	−0.55 (−0.90; −0.21)	*0.002*	−0.20 (−0.48; 0.08)	0.17
F-p-insulin (pmol/l)	−0.001 (−0.006; 0.004)	0.64	−0.005 (−0.01; 0.003)	0.22
HbA1c (%)	−0.45 (−0.96; 0.06)	0.09	−0.77 (−1.25; −0.29)	*0.002*
HOMA-IR	−0.04 (−0.18; 0.09)	0.52	−0.14 (−0.34; 0.06)	0.16
HOMA-B%	0.0003 (−0.004; 0.004)	0.86	−0.0007 (−0.005; 0.004)	0.76
FFA basal µmol/l^#^	0.0003 (−0.001; 0.002)	0.66	0.0002 (−0.0008; 0.001)	0.71
Total cholesterol (mmol/l)	0.09 (−0.07; 0.35)	0.26	−0.09 (−0.26; 0.07)	0.27
HDL (mmol/l)	0.19 (−0.31; 0.68)	0.45	0.08 (−0.42; 0.58)	0.76
LDL (mmol/l)	0.06 (−0.17; 0.29)	0.59	−0.10 (−0.29; 0.08)	0.27
VLDL (mmol/l)	−0.14 (−0.61; 0.33)	0.55	−0.30 (−1.18; 0.58)	0.50
Triglyceride (mmol/l)	−0.06 (−0.23; 0.11)	0.49	−0.11 (−0.51; 0.29)	0.59

Estimated change in telomere length is presented as β (95% CI). *Change in telomere length per 100 g change in lean or fat mass, ^#^n = 34 LBW and 37 NBW.

**Figure 2:**
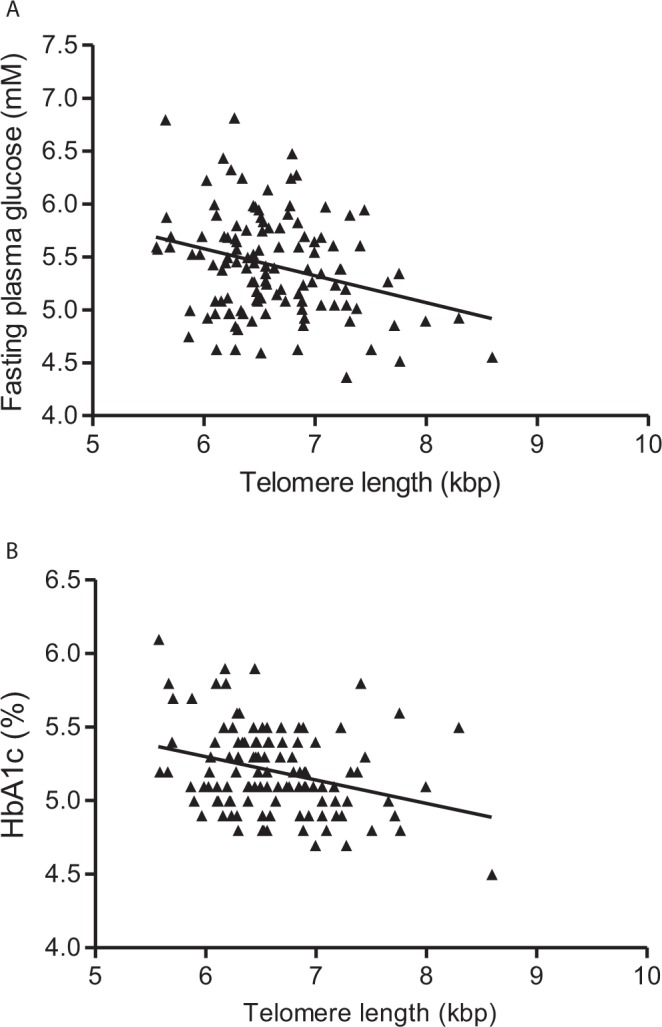
Associations between telomere length and fasting glucose (**A**) (n = 119) and HbA1c (**B**) (n = 115) in both LBW and NBW participants.

In the combined group of LBW and NBW individuals, there were inverse associations between telomere length and fasting glucose and telomere length and HbA1c (Fig. 2). When adjusting for birth weight group and cohort effect, significant negative associations between telomere length and fasting glucose (β = −0.32, P = 0.003) and telomere length and HbA1c (β = −0.60, P = 0.0008) were found. In the combined group there was borderline significant negative association with fat% (β = −0.01, P = 0.06) and borderline positive association with systolic blood pressure (β = 0.006, P = 0.07).

## Discussion

In the present study we showed that in young adulthood, there was no difference in leukocyte telomere length between individuals born with a LBW at term and age-matched individuals born with a normal birth weight. Interestingly, the telomere length was negatively associated with fasting plasma glucose levels and HbA1c when data from all the young healthy men was combined. Our current findings of elevated fasting glucose and insulin resistance among young men with LBW is in line with previous observations^12,13^ and underscore the importance of the intrauterine environment in the development of adult disease, including type 2 diabetes.

It is well-known that telomere attrition is associated with normal cellular aging^23^. More importantly, telomere attrition has been suggested to be a causal factor in the development of age-related diseases including type 2 diabetes^9^. Furthermore, studies have shown an impact of the intrauterine environment, as reflected by abnormal fetal growth, on telomere length in newborns^19,24^. In contrast, other studies have failed to show association between telomere length and birth weight, length or gestational age^25^ as well as to phenotype in adults aged from 16–51 years exposed to an adverse fetal environment^21,22,26^. A recent study found no difference in telomere shortening during a five-year period between adult individuals born small for gestational age (SGA) and appropriate for gestational age (AGA)^21^. This is further supported by a larger study including 49–51 year-old males and females, where no associations between blood telomere length and birth weight was observed^26^. Likewise, another study showed that young adult offspring of mothers with pre-gestational type 1 diabetes did not differ in terms of telomere length and DNA damage from BMI and weight-matched control individuals^22^. These studies are in support of our present findings of no difference in telomere length between two groups of young men distinguished by a large difference in birth weight. We cannot rule out that the telomere length of the current LBW subjects may have been shorter at birth and that time and lifestyle can have moderated and equalized this potential telomere difference. Nevertheless, based on previous and our findings of no persistent reduction of telomere length in adult LBW subjects, leucocyte telomere shortening is unlikely to play a central role in the association between fetal growth restriction and increased risk of developing type 2 diabetes later in life.

The rate of telomere attrition is highly variable^27^, and the telomere length can thus be modified by multiple factors from birth to adulthood. Shorter telomere has indeed been reported to be influenced by lifestyle factors including diet^28^, smoking^16^ and physical activity^29^. Besides environmental exposures, telomere lengths can be altered in response to social exposures as well. Interestingly, educational level^26^ and even parental responsiveness (describing parent and child interaction) seem to moderate the association between early adversity and telomere length, with higher parental responsiveness predicting longer telomeres^30^. In the current study we have no information on environmental factors which potentially could have affected the attrition of telomere length during childhood and adolescence.

Supporting our finding, several studies have showed an inverse correlation between telomere length and glucose levels. Recently, Rosa *et al*. showed in patient newly diagnosed with type 2 diabetes, that relative telomere length was inversely associated with fasting plasma glucose levels and HbA1c levels after adjustment for age, sex and body mass index^31^. Ahmad *et al*. found an inverse association between leucocyte telomere length and 2-h glucose concentrations in non-diabetic, middle-aged Caucasian adults^32^. The extent to which our findings of a negative association between leukocyte telomere length and fasting plasma glucose and HbA1c reflects a direct detrimental effect of early and minor changes in glucose metabolism on senescence at a cellular level, or whether this occurs as a result of confounding from associated anthropometric or lifestyle factors such as diet or physical activity is unknown. We have in a recent study showed that telomere length measured at the age of 9–16 years in offspring of mothers GDM and control offspring was significantly associated with fasting insulin levels and HOMA-IR among the girls, whereas no association was observed among the boys^20^, indicating a potential different influence of gender on these associations. In the current study (which only included males), subjects with birth weights at the upper 10% range were excluded to minimize risk of confounding from the influence of gestational diabetes. In another cross-sectional study examining healthy individuals at 8–80 years of age, an inversely association between fasting glucose and telomere length was found. However, after backward selection, fasting glucose was no longer significantly associated with telomere length whereas adiposity measures including both higher total and abdominal adiposity were significantly associated to shorter telomere length^33^. In the present study we found a tendency to an association between a higher fat percentage and shorter telomere length. Furthermore, telomere length at birth has been positively associated with lean mass and fat mass in newborns but only to lean mass at 12 months^34^, supporting that body composition plays a role in telomere shortening.

A meta-analysis showed an inverse association between leucocyte telomere length and risk of coronary heart disease^35^. In the present study, we found a significant positive association between telomere length and systolic blood pressure in LBW subjects, which was however, not significant in the pooled data set. This is supported by a study in adult females were no association between telomere length and blood pressure was found^36^ and further by a study were no association between telomere length and the risk of incident atrial fibrillation was observed^37^. Thus, more studies are needed to clarify the relationship between telomere length and cardiometabolic risk factors.

The strength of the current study includes the homogeneity of the study population as all participants were recruited according to standardized criteria and studied prior to development of diseases. Age is known to affect telomere length, and the current study population was young, with an age ranging from 21–25 years, reducing potential bias. Knowing that shorter telomere is associated with male gender^6^ and Caucasian race, this study included only men of Caucasian ethnicity reducing the variability within the groups. The participants were born in and still residing in the Greater Copenhagen area ensuring demographic similarity. Since a family history of type 2 diabetes is a risk factor for developing the disease in itself, the exclusion criteria included diabetes in the family, in order to rule out the possibility of major genetic confounding. Also, obese subjects with a BMI greater than 30 kg/m^2^ and those with a self-reported high physical activity level were excluded to avoid studying metabolic changes secondary to either obesity or a high level of physical activity.

The homogeneity of the study population is also a possible weakness since results are not necessarily generalizable. Moreover, it could be that resourceful individuals are more likely to participate in time-consuming studies and may represent the healthiest part of the population.

In the present study a qPCR high throughput method was used allowing for a large sample size to be analyzed that would not have been feasible using other methods such as Southern blotting. Unfortunately, cord blood was not available in the present cohort to allow examination of white cell telomere shortening over time, and likewise data on lifestyle, social status and other factors known to influence telomere attrition over time was not available. The present study is a cross-sectional study and to rule out the long-term potential impact of shorter telomeres, longitudinal studies examine telomere shortening over time and its associations with metabolic and adiposity phenotypes are needed. Since leukocyte telomere length has been suggested as a proxy for muscle telomere length^32^ and considering our findings of an association between telomere length and fasting glucose and HbA1c it would be highly interesting to examine telomere length in tissues involved in development of type 2 diabetes such as muscle, adipose tissue and liver. It is indeed possible, that telomere length in other more metabolically relevant tissues are not the same as in blood and furthermore, considering that some tissues are more vulnerable to oxidative stress than others, we might have seen an impact of the intrauterine environment on telomere length in tissues such as muscle or pancreatic tissues, if they had been available. Finally, we cannot exclude the possibility that the lack of finding an association between telomere length and birth weight is due to a small sample size, though studies of similar number of adult participants are in support of our finding, as they have also reported a lack of association^21,22,25,26^. However, the current study may not be fully powered to detect differences in the subgroup analyses.

In conclusion, we found no difference in telomere length between young, healthy men born with a LBW compared to NBW individuals. In support of the existing literature, we found that telomere length is significantly negatively associated with fasting blood glucose level within the normal range. Since white blood cells are suitable for providing information on biomarkers, but probably not the best for providing insight into biology, further validation studies are needed that includes both blood and samples from metabolic active tissue.

## Research Design and Methods

### Participants

One hundred and twenty young, lean and healthy Caucasian males aged 21–25 years were included in a cross-sectional study. All individuals were singletons and born at term (weeks 39–41) in 1980–1983 in Copenhagen County and were recruited from the Danish Medical Birth Registry according to birth weight and identical inclusion/exclusion criteria. Half of the subjects had a birth weight below the 10^th^ percentile for gestational age (LBW) and the other half had a birth weight in the upper normal range (50–90^th^ percentile) defined as normal birth weight (NBW). Participants with diabetes or with family members with known diabetes were excluded. Furthermore, a BMI ≥ 30 kg/m^2^, >10 hr exercise/week (self-reported) and an intake of medication known to affect glucose and lipid metabolism did also exclude individuals from participation.

Participants have been included in studies performed at Steno Diabetes Center, Gentofte, Denmark to determine the impact of the intrauterine environment and low birth weight on development of type 2 diabetes later in life, and have been extensively metabolically characterized according to separate protocols^12,38–40^. The association between LBW and cardiometabolic traits have been demonstrated and published previously^12,38–40^, however data are included in the current paper to describe the overall metabolic phenotype of the combined cohort and to associate it with telomere length.

To alleviate possible confounding of the results by factors such as alcohol, diet and physical activity, all participants were subjected to identical standardization including criteria for diet and alcohol intake and amount of physical activity 3 days prior to the examination.

Protocols were approved by the regional ethics committee of the Capital Region of Denmark, and procedures were performed according to the principles of The Helsinki Declaration. All subjects have received thorough written and oral information before giving written informed consent.

### Experimental protocols

Detailed descriptions of the study procedures have previously been reported^12,38–40^. All 120 subjects underwent identical standardized blood testing after an overnight fast, anthropometric and blood pressure measurements, a Dual-energy X-ray Absorptiometry (DXA) scan and determination of leukocyte telomere length. All blood samples were drawn from a polyethylene catheter placed in the antecubital vein and blood samples for plasma insulin were centrifuged immediately at 4 °C and stored at −80 °C prior to analysis. Plasma insulin, glucose, free fatty acids, leucocytes, HbA1c and serum concentrations of triglycerides, LDL and HDL cholesterols were analyzed by standard assays at Steno Diabetes Center, Gentofte, Denmark. Body composition was assessed by DXA (Lunar Radiation and Lunar Prodigy Advance, GE-healthcare, Madison, WI, USA). The homeostatic model assessment (HOMA) of insulin resistance (HOMA-IR) and beta cell function (HOMA-B%) were calculated according to Matthews *et al*.^41^. All methods were carried out in accordance with the relevant guidelines and regulations.

### Leukocyte telomere length

Genomic DNA from blood was isolated and purified using QIAamp DNA Blood Midi and QIAamp DNA Tissue Mini kits from Qiagen Inc. (QIAGEN Inc. 27220 Turnberry Lane, Valencia, CA 91355). The DNA concentration and purity were measured by spectrophotometry.

Telomere length was determined using a validated quantitative polymerase chain reaction (qPCR) based analysis compared to the reference gene 36B4 as described previously^42^. In brief, the method included amplifying the telomeres of the experimental DNA against a single copy gene and comparing the ratio of telomere to single copy gene concentration to that of a reference DNA of known telomere length. Reference DNA was isolated from a culture of human vascular smooth muscle cells and its length determined by Southern blots. Both telomere primers and PCR master mixes were designed and prepared using Applied Biosystems reagents, as previously described^42^. Primer concentrations were: Tel.(1) 270 nM, Tel.(2) 900 nM, 36B4(1) 300 nM 36B4(2) 500 nM. The primer sequences 7 were (5′–3′): Tel.(1) GGTTTTTGAGGGTGAGGGTGAGGGTGAGGGTGAGGGT, Tel.(2) TCCCGACTATCCCT-ATCCCTATCCCTATCCCTATCCCTA, 36B4(1) CAGCAAGTGGGAAGGTGTAATCC, 36B4(2) CCCATTCTATCATCAACGGGTACAA. qPCR was performed in 0.1 ml tubes on a Rotor-Gene™ 6000 QPCR thermocycler (Corbett Life Science, PO Box 435, Concorde, NSW 2137, Australia) with 15 μl of master mix and 5 μl of sample DNA per tube. Reference DNA was serially diluted 1:2, 6 times in water from 5 ng/μl to give a standard curve and 5 μl of each dilution was used per reaction. Sample DNA was diluted to 1 ng/μl and 5 μl used per reaction. Standard curves generated from the reference DNA were used to derive absolute telomere length from all study participants. Analysis of both telomere length and 36B4 in experimental samples were run in triplicate and both telomere and 36B4 amplifications were performed on the same run. Average ratios of telomere to 36B4 concentration were calculated from the triplicates of each experimental sample and compared to the ratio for the reference DNA to derive telomere length^43^. Telomere length is expressed as absolute telomere length (kilo base pairs, kbp) as it allows for a more direct comparison of results between other experiments and different cell types.

### Statistical analyses

All analyses were performed using SAS 9.4 statistical software (SAS institute, Cary, NC, USA) and p ≤ 0.05 was considered statistically significant. Normally distributed data were described as mean (±standard deviation, (SD)) whereas median and interquartile range was used for skewed outcomes. Differences in anthropometric, metabolic and body composition outcomes between LBW and NBW subjects were analyzed using Student’s t-test for normally distributed data and Wilcoxon test for non-normally distributed data. Associations between telomere length as outcome variable and phenotypic characteristics as exposure variables were examined by linear regression models. After ensuring that the residuals were normally distributed, we calculated β-coefficients and 95% confidence interval (CI). Since the study participants include subjects from 4 different clinical studies, the regression models were adjusted for cohort effect when analyzed separately in LBW and NBW subjects (Table 2) and for cohort effect and birth weight group when analyzed together.

## Data Availability

Data generated in the present study are available from the corresponding author upon reasonable request.

## References

[1] Blackburn EH (1991). Structure and function of telomeres. Nature.

[2] Mather KA, Jorm AF, Parslow RA, Christensen H (2011). Is Telomere Length a Biomarker of Aging? A Review. Journals Gerontol. Ser. A Biol. Sci. Med. Sci..

[3] Harley CB, Futcher AB, Greider CW (1990). Full-text. Nature.

[4] Sanders, J. L. & Newman, A. B. Telomere Length in Epidemiology: A Biomarker of Aging, Age-Related Disease, Both, or Neither? *Epidemiol. Rev* **35**, 112–31 (2013). 10.1093/epirev/mxs008 PMC4707879 23302541

[5] Frenck RW, Blackburn EH, Shannon KM (1998). The rate of telomere sequence loss in human leukocytes varies with age. Proc. Natl. Acad. Sci. USA.

[6] Gardner M (2014). Gender and telomere length: Systematic review and meta-analysis. Exp. Gerontol..

[7] Bekaert Sofie, De Meyer Tim, Rietzschel Ernst R., De Buyzere Marc L., De Bacquer Dirk, Langlois Michel, Segers Patrick, Cooman Luc, Van Damme Piet, Cassiman Peter, Van Criekinge Wim, Verdonck Pascal, De Backer Guy G., Gillebert Thierry C., Van Oostveldt Patrick (2007). Telomere length and cardiovascular risk factors in a middle-aged population free of overt cardiovascular disease. Aging Cell.

[8] Demissie S., Levy D., Benjamin E. J., Cupples L. A., Gardner J. P., Herbert A., Kimura M., Larson M. G., Meigs J. B., Keaney J. F., Aviv A. (2006). Insulin resistance, oxidative stress, hypertension, and leukocyte telomere length in men from the Framingham Heart Study. Aging Cell.

[9] Willeit P (2014). Leucocyte Telomere Length and Risk of Type 2 Diabetes Mellitus: New Prospective Cohort Study and Literature-Based Meta-Analysis. PLoS One.

[10] D’Mello MJJ (2015). Association Between Shortened Leukocyte Telomere Length and Cardiometabolic OutcomesCLINICAL PERSPECTIVE. Circ. Cardiovasc. Genet..

[11] Hales CN, Barker DJ (2001). The thrifty phenotype hypothesis. British medical bulletin.

[12] Brøns C (2008). Mitochondrial function in skeletal muscle is normal and unrelated to insulin action in young men born with low birth weight. J. Clin. Endocrinol. Metab..

[13] Hermann T. S., Rask-Madsen C., Ihlemann N., Domínguez H., Jensen C. B., Storgaard H., Vaag A. A., Kober L., Torp-Pedersen C. (2003). Normal Insulin-Stimulated Endothelial Function and Impaired Insulin-Stimulated Muscle Glucose Uptake in Young Adults with Low Birth Weight. The Journal of Clinical Endocrinology & Metabolism.

[14] Brøns C (2012). Effects of high-fat overfeeding on mitochondrial function, glucose and fat metabolism, and adipokine levels in low-birth-weight subjects. Am. J. Physiol. Metab..

[15] Clausen TD (2008). High prevalence of type 2 diabetes and pre-diabetes in adult offspring of women with gestational diabetes mellitus or type 1 diabetes: the role of intrauterine hyperglycemia. Diabetes Care.

[16] Salihu Hamisu M., Pradhan Anupam, King Lindsey, Paothong Arnut, Nwoga Chiaka, Marty Phillip J., Whiteman Valerie (2015). Impact of intrauterine tobacco exposure on fetal telomere length. American Journal of Obstetrics and Gynecology.

[17] Entringer S (2013). Maternal psychosocial stress during pregnancy is associated with newborn leukocyte telomere length. Am. J. Obstet. Gynecol..

[18] Strohmaier J (2015). Low Birth Weight in MZ Twins Discordant for Birth Weight is Associated with Shorter Telomere Length and lower IQ, but not Anxiety/Depression in Later Life. Twin Res. Hum. Genet..

[19] Lee S-P, Hande P, Yeo GS, Tan E-C (2017). Correlation of cord blood telomere length with birth weight. BMC Res. Notes.

[20] Hjort Line, Vryer Regan, Grunnet Louise G., Burgner David, Olsen Sjurdur F., Saffery Richard, Vaag Allan (2018). Telomere length is reduced in 9- to 16-year-old girls exposed to gestational diabetes in utero. Diabetologia.

[21] de Melo AS (2017). The telomere attrition rate is not accelerated in women born small for gestational age: A birth cohort study. Gene.

[22] Cross JA (2009). Absence of telomere shortening and oxidative DNA damage in the young adult offspring of women with pre-gestational type 1 diabetes. Diabetologia.

[23] Mayer S (2006). Sex-specific telomere length profiles and age-dependent erosion dynamics of individual chromosome arms in humans. Cytogenet. Genome Res..

[24] Tellechea M (2015). Telomere length in the two extremes of abnormal fetal growth and the programming effect of maternal arterial hypertension. Sci. Rep..

[25] Kajantie E (2012). No association between body size at birth and leucocyte telomere length in adult life–evidence from three cohort studies. Int. J. Epidemiol..

[26] Pearce MS (2012). Childhood Growth, IQ and Education as Predictors of White Blood Cell Telomere Length at Age 49–51 Years: The Newcastle Thousand Families Study. PLoS One.

[27] Aviv A (2009). Leukocyte telomere dynamics: longitudinal findings among young adults in the Bogalusa Heart Study. Am. J. Epidemiol..

[28] Zhou M (2015). Influence of diet on leukocyte telomere length, markers of inflammation and oxidative stress in individuals with varied glucose tolerance: a Chinese population study. Nutr. J..

[29] Magi F (2018). Telomere length is independently associated with age, oxidative biomarkers, and sport training in skeletal muscle of healthy adult males. Free Radic. Res..

[30] Asok A, Bernard K, Roth TL, Rosen JB, Dozier M (2013). Parental responsiveness moderates the association between early-life stress and reduced telomere length. Dev. Psychopathol..

[31] Rosa ECCC (2018). Leukocyte telomere length correlates with glucose control in adults with recently diagnosed type 2 diabetes. Diabetes Res. Clin. Pract..

[32] Ahmad S., Heraclides A., Sun Q., Elgzyri T., Rönn T., Ling C., Isomaa B., Eriksson K.-F., Groop L., Franks P. W., Hansson O. (2012). Telomere length in blood and skeletal muscle in relation to measures of glycaemia and insulinaemia. Diabetic Medicine.

[33] Lee M, Martin H, Firpo MA, Demerath EW (2011). Inverse association between adiposity and telomere length: The Fels Longitudinal Study. Am. J. Hum. Biol..

[34] de Zegher F, Díaz M, Lopez-Bermejo A, Ibáñez L (2017). Recognition of a sequence: more growth before birth, longer telomeres at birth, more lean mass after birth. Pediatr. Obes..

[35] Haycock PC (2014). Leucocyte telomere length and risk of cardiovascular disease: systematic review and meta-analysis. BMJ.

[36] Aydos, S. E. & Tükün, A. Does telomere length affect blood pressure? *Adv. Ther*. **24**, 269–72 (2007). 10.1007/BF02849894 17565916

[37] Staerk L (2017). Association Between Leukocyte Telomere Length and the Risk of Incident Atrial Fibrillation: The Framingham Heart Study. J. Am. Heart Assoc..

[38] Jensen C. B., Storgaard H., Dela F., Holst J. J., Madsbad S., Vaag A. A. (2002). Early Differential Defects of Insulin Secretion and Action in 19-Year-Old Caucasian Men Who Had Low Birth Weight. Diabetes.

[39] Schou Jakob Hagen, Pilgaard Kasper, Vilsbøll Tina, Jensen Christine B., Deacon Carolyn F., Holst Jens Juul, Vølund Aage, Madsbad Sten, Vaag Allan A. (2005). Normal Secretion and Action of the Gut Incretin Hormones Glucagon-Like Peptide-1 and Glucose-Dependent Insulinotropic Polypeptide in Young Men with Low Birth Weight. The Journal of Clinical Endocrinology & Metabolism.

[40] Alibegovic AC (2010). Increased rate of whole body lipolysis before and after 9 days of bed rest in healthy young men born with low birth weight. Am. J. Physiol Endocrinol. Metab.

[41] Matthews DR (1985). Homeostasis model assessment: insulin resistance and beta-cell function from fasting plasma glucose and insulin concentrations in man. Diabetologia.

[42] Calvert PA (2011). Leukocyte Telomere Length Is Associated With High-Risk Plaques on Virtual Histology Intravascular Ultrasound and Increased Proinflammatory Activity. Arterioscler. Thromb. Vasc. Biol..

[43] O’Callaghan NJ, Fenech M (2011). A quantitative PCR method for measuring absolute telomere length. Biol. Proced. Online.

